# A Soldier or Sailor No Better Than His Teeth

**Published:** 1917-03

**Authors:** 


### A SOLDIER OR SAILOR NO BETTER THAN HIS TEETH.

### DR. GRADY'S PREPAREDNESS IDEA IS SET FORTH AT THE FORSYTH DENTAL INFIRMARY, BOSTON.

Writing in "Frocks and Frills" for the *Baltimore Sun* in a recent edition of that paper, Miss Emily E. Lantz, Editor of "Frocks and Frills" has the following of local interest:

A SOLDIER OR SAILOR IS NO BETTER THAN HIS TEETH.

While meditating upon the things Baltimore needs for 1917, a news paragraph catches the eye. It is in effect that in Boston, Mass., on January 20, 1917, members of the dental profession will present a silver loving cup to Mr. Thomas A. Forsyth, who has given to the city in the Forsyth Dental Infirmary for Children the first institution of its kind in the world. It is an infirmary where the teeth of children are examined and treated free of charge and where the public is taught the close relation between sound teeth and health and instructed how to take care of the former. Oliver Wendell Holmes has said: "The dental profession has established and prolonged the reign of beauty; it has added to the charms of social intercourse and lent perfection to the accents of eloquence; it has taken from old age its most unwelcome feature, and lengthened enjoyable human life far beyond the limit of years when the toothless and purblind patriarch might well exclaim, 'I have no pleasure in them.'"

In addition to the gift of the building, an endowment of $2,000,000 will be provided for the maintenance of the Forsyth Dental Infirmary. Outwardly, and in the interior, the building is architecturally beautiful. It is equipped with every facility that dental science demands, and there is a garden where children, awaiting treatment, may play. There is also a waiting room, airy and cheerful where decorative panels of the walls picture Browning's story of the Pied Piper of Hamelin, Washington Irving's tale of Rip Van Winkle, the classic narrative of the Argosy and the Golden Fleece and the diverting folk tales of the Dorchester Giant.

In this great training school for physical national preparedness Maryland has a part because the subject of oral hygiene in relation to school children was first introduced in 1900 by Dr. Richard Grady, formerly of Baltimore, and now dental surgeon of the United States Naval Academy at Annapolis. Dr. Grady was also the organizer and for many years the director of the Baltimore Polytechnic Institute, the first school devoted to manual training in America. Among his dental associates he is known as the 'father of the national mouth hygiene movement' and quite fittingly he delivered at the Forsyth Infirmary on December 10 the first of the course of free lectures to be given by eminent men upon subjects relating to public health.

Dr. Grady's preparedness idea is that no soldier nor sailor is better than his teeth. His dental creed is, 'Good teeth, good health,' with the logical explanation:

Without good teeth, there can not be thorough mastication.

Without thorough mastication, there can not be perfect digestion.

Without perfect digestion, there can not be proper assimilation.

Without proper assimilation, there can not be nutrition.

Without nutrition, there can not be health.

Without health, what is life?

Hence the paramount importance of the teeth.

It is infinitely more vital to teach children this creed than the free-verse rendering of the immortal tale concerning the ''House that Jack Built.''

The public is thoroughly alive to its duty toward the child in developing its mind, yet of equal importance is the development of its body. The child with neglected teeth suffers inferior physical development. Baltimore requires no systematic examination of the teeth of children admitted to the public schools other than that incident to the physical examination made by health officers, and there are free dental clinics to which attendance is voluntary. Yet here is a dental infirmary already provided in Boston. It is said in dental circles that through the munificence of a private citizen, Rochester, N. Y., is soon to receive an endowment of $1,500,000 to establish a Rochester Dental Dispensary, and that a California believer in national preparedness is to endow a similar institution in the West.

